# Supplementary material for: Coupling of remote alternating-access transport mechanisms for protons and substrates in the multidrug efflux pump AcrB
Source: eLife. 2014 Sep 19;3:e03145. doi: 10.7554/eLife.03145 (PMC4359379; doi:10.7554/eLife.03145)
Supplement: Supplementary file 1. — DOI: http://dx.doi.org/10.7554/eLife.03145.029 [file elife03145s001.docx]

|  | **PN1+PC2 v PC1+PN2 PC1+PN2 v PC1+PN2 PN1+PC2 v PN1+PC2** | | |  | **PN1 v PC1 PC1 v PC1 PN1 v PN1** | | |  | **PC2 v PN2  PN2 v PN2 PC2 v PC2** | | |
| --- | --- | --- | --- | --- | --- | --- | --- | --- | --- | --- | --- |
|  | L | T | O |  | L | T | O |  | L | T | O |
| L | 4.9 | **2.0** | 0.4 |  | 2.0 | 0.5 | 0.2 |  | 2.0 | 0.3 | 0.3 |
| T | 0.4 | 3.8 | **1.8** |  | 0.5 | 2.1 | 0.4 |  | 0.1 | 2.0 | 0.3 |
| O | 0.5 | 0.4 | 4.6 |  | 0.4 | 0.4 | 2.0 |  | 0.3 | 0.3 | 2.0 |

**Supplementary file 1.** Structural variations in the porter domain of the periplasmic region of wildtype AcrB, in the L, T and O states. The porter domain in each AcrB protomer consists of two repeats, referred to as PN1+PC2 and PC1+PN2, each of which is a tandem of two α/β sub-domains. Each α/β domain consists of a sheet of 4 β-strands (PN1: residues 41 to 49, 76 to 83, 86 to 94 and 126 to 131; PC2: residues 679 to 685, 714 to 718, 824 to 830, 856 to 860; PC1: residues 570 to 578, 607 to 614, 623 to 631 and 662 to 667; PN2: residues 137 to 143, 173 to 177, 286 to 292, 322 to 326), flanked by a pair of α-helices (PN1: 55 to 69, 102 to 117; PC2: 693 to 709, 836 to 851; PC1: 584 to 598, 643 to 658; PN2: 153 to 169, 298 to 313). The two β-sheets in each repeat are connected by a common β-strand (PN1+PC2: residues 277 to 286; PC1+PN2: residues 815-824). The differences between two structures or conformational states are quantified in terms of the root-mean-squared difference (RMSD) between them, after least-squares fitting. Only the backbone (N, Cα, C, O) of secondary-structure elements is considered; all values are given in Å. Comparisons are made of the complete two-domain repeat (left), the first α/β domain in each repeat (middle), or the second α/β domain in each repeat (right). In each case, off-diagonal values are comparisons of a given element in different conformational states of the AcrB protomer, while diagonal values are comparisons of two different repeats in the same conformational state. This analysis shows that the PN1+PC2 repeat is largely rigid throughout the conformational cycle, while PC1+PN2 varies significantly in the L to T and T to O transitions (bold). This is due to a change in the relative orientation of PC1 and PN2, and not to a change in their internal structure. The structures of PN1+PC2 and PC1+PN2 differ significantly in all states.
