# Supplementary material for: Coupling of remote alternating-access transport mechanisms for protons and substrates in the multidrug efflux pump AcrB
Source: eLife. 2014 Sep 19;3:e03145. doi: 10.7554/eLife.03145 (PMC4359379; doi:10.7554/eLife.03145)
Supplement: Supplementary file 2. — DOI: http://dx.doi.org/10.7554/eLife.03145.030 [file elife03145s002.docx]

|  | D407N | D408N | K940A | R971A |
| --- | --- | --- | --- | --- |
| Wavelength (Å) | 1.0 | 1.0 | 1.0 | 0.8 |
| Resolution range (Å) | 48.93 - 2.298  (2.38 - 2.298) | 48.8 - 2.099  (2.174 - 2.099) | 48.99 - 2.0  (2.071 - 2.0) | 48.9 - 2.2  (2.279 - 2.2) |
| Space group | P 21 21 21 | P 21 21 21 | P 21 21 21 | P 21 21 21 |
| Unit cell | 145.59 161.59 245.97  90 90 90 | 145.88 161.16 245.29  90 90 90 | 145.91 162.32 245.81  90 90 90 | 145.79 160.77 246.45  90 90 90 |
| Total reflections | 1888523 (146962) | 2386764 (155795) | 2481254 (103921) | 2457131 (199146) |
| Unique reflections | 256709 (25399) | 332174 (30871) | 349404 (20567) | 290078 (27494) |
| Multiplicity | 7.4 (5.8) | 7.2 (5.0) | 7.1 (5.1) | 8.5 (7.2) |
| Completeness (%) | 99.95 (99.68) | 99.16 (92.75) | 89.38 (53.12) | 99.35 (94.90) |
| Mean I/sigma(I) | 11.12 (0.84) | 9.63 (0.78) | 11.04 (0.50) | 7.74 (0.80) |
| Wilson B-factor | 37.65 | 28.67 | 27.85 | 28.82 |
| R-merge | 0.2026 (2.398) | 0.1974 (2.004) | 0.2019 (3.32) | 0.3129 (2.726) |
| R-meas | 0.218 | 0.2128 | 0.2176 | 0.3336 |
| CC1/2 | 0.997 (0.293) | 0.996 (0.244) | 0.996 (0.143) | 0.989 (0.22) |
| CC* | 0.999 (0.673) | 0.999 (0.626) | 0.999 (0.5) | 0.997 (0.601) |
| R-work | 0.1950 (0.3488) | 0.1960 (0.3538) | 0.1965 (0.3650) | 0.2012 (0.3349) |
| R-free | 0.2366 (0.3719) | 0.2390 (0.3788) | 0.2363 (0.3795) | 0.2418 (0.3711) |
| Number of non-hydrogen atoms | 27495 | 28217 | 28430 | 28533 |
| Macromolecules | 25944 | 25944 | 25933 | 25927 |
| Ligands | 208 | 161 | 138 | 237 |
| Water | 1343 | 2112 | 2359 | 2369 |
| Protein residues | 3418 | 3418 | 3418 | 3418 |
| RMS (bonds) | 0.070 | 0.063 | 0.056 | 0.082 |
| RMS (angles) | 1.30 | 1.29 | 1.28 | 1.39 |
| Ramachandran favored (%) | 97 | 97 | 97 | 96 |
| Ramachandran outliers (%) | 0.44 | 0.59 | 0.62 | 0.62 |
| Clashscore | 6.83 | 6.24 | 6.59 | 8.08 |
| Average B-factor | 51.80 | 40.60 | 41.50 | 39.40 |
| macromolecules | 51.70 | 40.10 | 40.90 | 38.90 |
| ligands | 86.30 | 63.10 | 63.00 | 73.50 |
| solvent | 47.80 | 45.30 | 47.20 | 41.80 |

**Supplementary file 2.** Data collection and refinement statistics for the crystal structures of AcrB mutants D407N, D408N, R971A, and K940A. Statistics for the highest-resolution shell are shown in parentheses.
