# Supplementary material for: Coupling of remote alternating-access transport mechanisms for protons and substrates in the multidrug efflux pump AcrB
Source: eLife. 2014 Sep 19;3:e03145. doi: 10.7554/eLife.03145 (PMC4359379; doi:10.7554/eLife.03145)
Supplement: Supplementary file 3. — DOI: http://dx.doi.org/10.7554/eLife.03145.031 [file elife03145s003.docx]

|  | **WT vs. D407N** | | | **WT vs. D408N** | | | **WT vs. R971A** | | | **WT vs. K940A** | | |
| --- | --- | --- | --- | --- | --- | --- | --- | --- | --- | --- | --- | --- |
|  | L | T | O | L | T | O | L | T | O | L | T | O |
| **R1** | 0.4 | 0.6 | 0.3 | 0.4 | 0.4 | 0.2 | 0.6 | 0.8 | 0.2 | 0.4 | 0.2 | 0.2 |
| **R2** | 0.4 | 0.4 | 0.3 | 0.2 | 0.2 | 0.2 | 0.5 | 0.6 | 0.3 | 0.2 | 0.2 | 0.2 |
| **PN1+PC2** | 0.1 | 0.2 | 0.3 | 0.2 | 0.1 | 0.1 | 0.1 | 0.2 | 0.2 | 0.1 | 0.1 | 0.2 |
| **PC1+PN2** | 0.2 | 0.4 | 0.1 | 0.1 | 0.2 | 0.1 | 0.2 | 0.4 | 0.1 | 0.1 | 0.1 | 0.1 |

**Supplementary file 3A.** Differences in the structures of the repeats in the transmembrane and porter domains of AcrB, in wildtype vs. D407N, D408N, R971A, and K940A variants. The difference between analogous repeats (defined in **Supplementary file 1** and **Figure 2 – figure supplement 1**) in wildtype and variant forms of the protein is quantified by the RMSD (in Å) of the corresponding structures after least-square fitting. The data shows that the substitutions have little or no influence in the overall internal structure of the repeats.

|  | | **D407N** | | | **D408N** | | | **R971A** | | | **K940A** | | |
| --- | --- | --- | --- | --- | --- | --- | --- | --- | --- | --- | --- | --- | --- |
|  |  | L | T | O | L | T | O | L | T | O | L | T | O |
| **WT** | L | **0.3** | 3.0 | 4.5 | **0.1** | 3.0 | 4.5 | **0.3** | 3.0 | 4.5 | **0.1** | 3.2 | 4.5 |
|  | T | 3.3 | **0.7** | 5.7 | 3.2 | **0.2** | 5.7 | 3.2 | **0.6** | 5.7 | 3.1 | **0.2** | 5.7 |
|  | O | 4.4 | 5.5 | **0.2** | 4.5 | 5.7 | **0.1** | 4.4 | 5.6 | **0.2** | 4.6 | 5.8 | **0.2** |

**Supplementary file 3B.** Differences in the orientation of repeat PN1+PC2 relative to PC1+PN2, in wildtype vs. D407N, D408N, R971A, and K940A, in the L, T and O states. To quantify the changes in the relative orientation of PN1+PC2 and PC1+PN2 upon mutation, the PC1+PN2 repeats in wildtype and variant AcrB were superimposed, and the RMSD between the corresponding PN1+PC2 repeats was then computed (all values in Å). The data shows that none of the substitutions has a noticeable influence in the orientation of the two porter-domain repeats. That is, in the variant forms, the conformation of the repeats in each protomer closely resembles their equivalent in the wildtype form (highlighted diagonal values).

|  | | **D407N** | | | **D408N** | | | **R971A** | | | **K940A** | | |
| --- | --- | --- | --- | --- | --- | --- | --- | --- | --- | --- | --- | --- | --- |
|  |  | L | T | O | L | T | O | L | T | O | L | T | O |
| **WT** | L | **1.4** | 3.1 | 2.5 | **0.5** | 1.1 | 3.0 | **2.5** | 3.8 | 2.7 | **0.6** | 1.6 | 2.7 |
|  | T | 2.8 | 1.7 | 1.7 | 2.0 | **0.6** | 2.1 | 3.6 | 2.4 | 1.8 | 1.1 | **0.2** | 1.8 |
|  | O | 3.9 | **1.6** | **0.7** | 3.4 | 2.4 | **0.2** | 4.4 | **1.6** | **0.5** | 2.7 | 2.0 | **0.4** |

**Supplementary file 3C.** Differences in the orientation of repeat R2 relative to R1, in wildtype vs. D407N, D408N, R971A, and K940A, in the L, T and O states. To quantify the changes in the relative orientation of R2 and R1 upon mutation, the R1 repeats in wildtype and variant AcrB were superimposed, and the RMSD between the corresponding R2 repeats was computed (all values in Å). The data shows that the D408N, K940A substitutions have little influence in this regard. That is, in the variant forms the conformation of the repeats in each protomer closely resembles their equivalent in the wildtype form (highlighted diagonal values). The D407N and R971A mutations, by contrast, alter the relative orientation of the repeats (though not their internal structure, as shown in Supplementary file 3A), in both the L and T states. Particularly in the T state, the mutated protomers are more similar to the wildtype O conformation than to the T conformation (highlighted off-diagonal values).
